# Age‐Related Deterioration of Bone Toughness Is Related to Diminishing Amount of Matrix Glycosaminoglycans (GAGs)

**DOI:** 10.1002/jbm4.10030

**Published:** 2018-02-18

**Authors:** Xiaodu Wang, Rui Hua, Abu Ahsan, Qingwen Ni, Yehong Huang, Sumin Gu, Jean X Jiang

**Affiliations:** ^1^ Department of Mechanical Engineering University of Texas at San Antonio San Antonio Texas; ^2^ Department of Biochemistry and Structural Biology University of Texas Health Science Center at San Antonio San Antonio Texas; ^3^ Department of Physics Texas A&M International University Laredo Texas

**Keywords:** BONE, PROTEOGLYCAN, TOUGHNESS, AGING, GLYCOSAMINOGLYCAN

## Abstract

Hydration status significantly affects the toughness of bone. In addition to the collagen phase, recent evidence shows that glycosaminoglycans (GAGs) of proteoglycans (PGs) in the extracellular matrix also play a pivotal role in regulating the tissue‐level hydration status of bone, thereby affecting the tissue‐level toughness of bone. In this study, we hypothesized that the amount of GAGs in bone matrix decreased with age and such changes would lead to reduction in bound water and subsequently result in a decrease in the tissue‐level toughness of bone. To test the hypothesis, nanoscratch tests were conducted to measure the tissue‐level toughness of human cadaveric bone specimens, which were procured only from male donors in three different age groups: young (aged 26 ± 6 years), mid‐aged (aged 52 ± 5 years), and elderly (aged 73 ± 5 years), with 6 donors in each group. Biochemical and histochemical assays were performed to determine the amount and major subtypes of GAGs and proteoglycans in bone matrix. In addition, low‐field nuclear magnetic resonance (NMR) measurements were implemented to determine bound water content in bone matrix. The results demonstrated that aging resulted in a statistically significant reduction (17%) of GAGs in bone matrix. Concurrently, a significant deterioration (20%) of tissue‐level toughness of bone with age was observed. Most importantly, the deteriorated tissue‐level toughness of bone was associated significantly with the age‐related reduction (40%) of bound water, which was partially induced by the decrease of GAGs in bone matrix. Furthermore, we identified that chondroitin sulfate (CS) was a major subtype of GAGs, and the amount of CS decreased with aging accompanied with a decrease of biglycan that is a major subtype of PGs in bone. The findings of this study suggest that reduction of GAGs in bone matrix is likely one of the molecular origins for age‐related deterioration of bone quality. © 2017 The Authors. *JBMR Plus* is published by Wiley Periodicals, Inc. on behalf of the American Society for Bone and Mineral Research.

## Introduction

Bone fragility fractures are a major health care concern for our rapidly growing aging population because of the high risk of long‐term disability and even mortality.^1^ Currently, bone mineral density (BMD) is commonly utilized by clinicians to predict the risk of bone fragility fractures.^2^, ^3^, ^4^ However, BMD alone is not ideally suitable for fracture risk assessments because bone fragility is not only induced by loss of BMD but also from detrimental changes of bone tissue at different hierarchies, including changes at ultrastructural and molecular levels.^5^, ^6^, ^7^, ^8^ Therefore, elucidating the ultrastructural and molecular origins of bone fragility becomes imperative for better prediction and prevention of such fractures.

Bone is composed of mineralized collagen fibrils embedded in an extrafibrillar matrix,^9^, ^10^ which has been shown to be an aggregate of mineral (carbonated apatite) nanocrystals and noncollagenous proteins (NCPs).^11^ In the past, the contribution of the mineral and collagen phases to bone mechanical competence has been extensively investigated.^12^, ^13^, ^14^, ^15^, ^16^, ^17^ However, it was not until recently that evidence started to emerge showing NCPs have direct effects on the mechanical behavior of bone.^18^, ^19^

In addition to type I collagen (90% of the organic phase) that serves as a major source of bound water in bone, NCPs comprise 10% of the organic phase of bone and also possess great capacity in attracting water molecules into bone matrix.^20^ Among NCPs, proteoglycans (PGs) are reported to exist outside of collagen fibrils with strong affinity to extrafibrillar minerals.^21^, ^22^ PGs contain abundant glycosaminoglycans (GAGs), which are highly negatively charged and possess a great potential of trapping and retaining water molecules in the matrix.^23^, ^24^ In fact, the similar role of GAGs has been well documented for different types of connective tissues, such as articulate cartilage and intervertebral disks.^25^, ^26^, ^27^ This feature is very important because water functions as a plasticizer that facilitates the plastic deformation of bone at both bulk^28^, ^29^, ^30^, ^31^ and ultrastructural levels.^32^

Previous evidence has shown that PGs play an important role in the mechanical behavior of bone. For instance, PGs have been found to regulate the hydrostatic and osmotic pressure, thus affecting the poroelastic behavior of dentine and bone tissues.^33^, ^34^ A recent study of our group demonstrates that loss of GAGs may significantly reduce the tissue‐level toughness of bone and such effects are most likely induced by the associated loss of bound water in bone matrix.^35^ Intriguingly, recent studies show that among the age‐related ultrastructural changes,^36^, ^37^, ^38^, ^39^ the loss of bound water in bone matrix with increasing age may directly lead to significant reduction of the toughness of bone.^40^, ^41^, ^42^, ^43^, ^44^ Based on the aforementioned evidence, it could be conjectured that changes in matrix GAGs may contribute to the age‐related deterioration of bone quality.

To this end, we hypothesized in the study that the amount of GAGs in bone matrix decreases with age, leading to loss of bound water in the bone matrix, and subsequently resulting in the deterioration of the tissue level toughness of bone. To test the hypothesis, biochemical and biomechanical assays were performed on human cadaveric bone samples from different age groups to determine whether the amount of GAGs, accompanied with loss of bound water in bone matrix, decreases with age, thus leading to the age‐related reduction of the tissue‐level toughness of bone. In addition, the major subtype(s) of GAGs and PGs in bone matrix was also examined.

## Materials and Methods

### Bone specimen preparation

Eighteen human cadaveric femurs procured from cortical bone samples were collected from a research tissue bank (National Disease Research Interchange, Philadelphia, PA, USA) in three different age groups: young (aged 26 ± 6 years), mid‐aged (aged 52 ± 5 years), and elderly (aged 73 ± 5 years). Six donors (*n* = 6) were included in each age group, with multiple specimens prepared from each donor, including one cube for nanoscratch test, bone powder samples for NMR test and biochemical assays of PGs/GAGs, and one slice for histochemical assays. Bone cubes and slices were cut using a precision low‐speed diamond saw (ISOMET 2000, Buehler, IL, USA) and then lapped and polished down to the final dimensions of 5.0 mm × 5.0 mm (about 800 μm thick). All of these samples were collected from the same quadrant (ie, anterior aspect) of mid‐diaphysis of the femurs.

### Histochemical assays for GAGs detection

Alcian blue staining is a semiquantitative method to measure GAG contents. Bone slices prepared as described above were used to verify relative differences for GAGs in bone samples from the different age groups. Alcian blue (Sigma‐Aldrich, St. Louis, MO, USA) assay was used on bone samples. Bone slices were incubated in 3% acetic acid for 3 minutes and then incubated in 1% Alcian blue, pH 2.5, for 1 hour at room temperature using a protocol modified from Luna and colleagues^45^ followed by destaining in a solution of ethanol/water/acetic acid at a ratio of 9:10:1 for 20 minutes.

### Measurement of the tissue‐level toughness of bone using a nanoscratch test

For nanoscratch tests, one bone cube was dissected from each femur. Then, the bone cubes were further lapped and polished down to the final dimensions (4.5 mm × 4.5 mm × 4.5 mm). To exclude the potential confounding effects from microstructural features (eg, Haversian canals, lacunae, cement lines, etc.), a nanoscratch approach was used to determine the tissue‐level toughness of bone. Briefly, the polished bone cubes were glued onto the holder in a custom‐designed chamber filled with PBS solution to ensure a wet condition throughout the test. The nanoscratch tests were performed circumferentially within individual lamellae of osteons on a Nano Indenter XP system (Agilent Technologies, Santa Clara, CA, USA) with a cube corner tip, following previously reported procedures.^35^, ^46^ Measurements were performed within lamellar layer on 6 randomly chosen newly formed osteons in the cross section for each test specimen to alleviate effects of possible variations caused by different biological tissue age and local heterogeneity in bone. The cross‐profile of the scratch groove was measured to estimate the width of the scratch groove. A constant penetration load was set to 5 mN and the scratch length was set to 20 µm for the nanoscratch test to ensure the scratch being conducted within a lamella. By doing so, the effect of microstructural heterogeneity of bone (eg, lamellar interface and lacunae) on the measurements could be circumvented. The tissue‐level toughness of bone was determined using the following equation,

(1) $$u_{s}=\frac{F_{t}L}{V}=\frac{F_{t}}{A}$$

where, *u_s_* is the nanoscratch toughness, *F_t_* is the lateral scratch force, *L* is the scratch length, *V* is the volume of the scratch groove, and *A* is the average cross‐sectional area of the scratch groove. *A* was estimated using the cross‐sectional and longitudinal profile of the scratch groove. The average of the 6 measurements was used as the tissue‐level toughness of each bone specimen.

### Quantification of the amount of GAGs in bone matrix

For the biochemical assays to determine the amount of GAGs, bone powders were prepared from different age groups by crushing bone samples into powders in liquid nitrogen. Using the powders, GAGs were isolated from the nonmineralized (eg, osteoids, membranes, and bone surfaces) and mineralized compartments in bone matrix, respectively, based on a modified protocol published in the literature.^47^, ^48^, ^49^ Briefly, cortical bone tissues were crushed to fine powders with the pestle in mortar filled with liquid nitrogen. The bone powders were first treated in lysis buffer I (4 M guanidine HCl, 0.05 M Tris, and 0.1 M 6‐aminocaproic acid, pH 7.4) at 4°C on the orbital shaker for 72 hours to remove GAGs from the nonmineralized compartment of bone. Then, the carefully rinsed bone powders were treated in lysis buffer II (4 M guanidine HCl, 0.5 M EDTA tetrasodium salt, 0.05 M Tris, and 0.1 M 6‐aminocaproic acid, pH 7.4) at 4°C on the orbital shaker for 72 hours to remove GAGs from the mineralized compartment of bone. The supernatant from each treatment was collected and the amount of GAGs was quantified by dimethylmethylene blue (DMMB) assay as follows: 50 μL samples and 200 μL DMMB mix (46 μM DMMB (Sigma), 40 mM NaCl, and 40 mM Glycine, adjusted pH to 3.0 with acetic acid) were pipetted into 96‐well plates and shaking the plate for 5 seconds. The absorbance was read at 525 nm wavelength. The amount of GAGs was determined by comparing with the standard curves generated using purified GAGs (Sigma) following a published protocol.^50^

### Low‐energy NMR measurement of bound water in bone matrix

The bound water was measured using a low‐field NMR technique.^43^, ^51^ Based on the T_2_ relaxation information, relative amount of freely mobile water, bound water, and lattice water were assessed by using NMR free induction decay (FID) measurement. Briefly, bone powders were dried at room temperature in vacuum for 10 hours to remove the excessive free water from the surface of the powders, which might result in large noises during NMR measurements. This drying time was selected to eliminate the noises based on the result of pilot experiments. The bone powder samples were weighted and the amount of bound water of the samples was measured using a low‐field NMR spectrometer (Bruker 20 MHz, Kontich, Belgium) at a proton frequency of 20 MHz for these measurements. 1H spin–spin (T_2_) relaxation profiles were obtained using the NMR CPMG, 90° [−τ − 180° − τ (echo)]n − TR, spin echo method, which has a 9.5 µs wide RF−90° pulse, τ of 1000 µs, and TR (sequence repetition rate) of 15 seconds. One thousand echoes were recorded (one scan with *n* = 1000) to obtain the T_2_ profile, with 64 scans used for the measurements. The FID signal was sampled and recorded at 2 µs intervals using a 9.5 µs wide 90° RF‐pulse for bound water measurements. For each FID profile, 1500 data points were acquired in one scan (an approximate 3 ms delay window). An inversion relaxation technique was used to invert FID data to a T_2_ relaxation distribution spectrum. The relative amount of bound water was then estimated as the ratio of the total intensity of bound water signal with respect to the total intensity of the solid form water signal (representative of bone mass because in these sample measurements, the solid form proton signal intensities were correlated with the sample mass weights) of each sample.

### Identification of subtypes of GAGs and PGs in bone matrix

We measured the bone volume using a top‐loading electronic balance with the density determination kit (Mettler Toledo, Columbus, OH, USA). The bone sample was first weighed in air and then immersed in water. The bone volume was calculated according to the Archimedean Principle using the formula as below:

V=(A−B)/ρ∘

where, A = weight of bone sample in air; B = weight of bone sample when immersed in water; and ρ∘ = density of water at a given temperature T.

The bone lysates with identical bone volume from different aging groups were loaded on agarose gels. Therefore, the loading controls were calibrated based on per volume (mm^3^) of each sample. GAGs isolated from nonmineralized and mineralized bone samples as well as GAGs standards, heparin sulfates (HS), dermatan sulfates (DS), and chondroitin sulfates (CS) were separated by 0.9% agarose gel electrophoresis in 1,3‐propanediamine acetate buffer (50 mM, pH 9.0) for 90 minutes at 80V. After the electrophoresis, the GAGs were fixed using 0.2% (w/v) cetyl‐trimethyl‐ammonium bromide (CTAB) solution for 1 hour at room temperature, and then the gels were dried. Gels were stained with 0.1% (w/v) toluidine blue prepared in solution of 50% (v/v) ethanol, 49% (v/v) water, and 1% (v/v) water, and destained with the same solution without toluidine blue.

Western blotting assay was conducted to identify PGs in the mineralized matrix of human bone. Supernatants isolated from mineralized matrix of human cortical bone were treated with or without Protein Deglycosylation Enzyme Mix (New England Biolabs P6044, Ipswich, MA, USA) for 1 hour at 37°C. The Protein Deglycosylation Enzyme Mix contains PNGase F, O‐glycosidase, neuraminidase, β1‐4‐galactosidase, and β‐N‐acetylglucosaminidase, which lead to complete deglycosylation. Equal amount of proteins determined by microBCA assay was loaded on precast 7.5% SDS‐polyacrylamide gel electrophoresis (PAGE) (Bio‐Rad, Hercules, CA, USA), transferred to nitrocellular membrane and immunoblotted with anti‐biglycan antibody (Santa Cruz Biotechnology, Dallas, TX, USA) (1:500 dilution).

### Statistical analysis

Bootstrapping was employed in all statistical analyses using SPSS statistical analysis package (IBM Corp., Armonk, NY, USA). Thus, requirements of conventional statistical analyses (eg, normality and equal variance) were no longer necessary. One‐factor ANOVA analyses were performed to determine the effect of age on the tissue‐level toughness of bone, the amount of GAGs, and the bound water content. Then, multiple comparisons (Fisher's PLSD) were implemented to determine the statistical differences between the age groups. Moreover, Pearson correlation analyses were performed to determine the correlation among the tissue‐level toughness, GAGs, and bound water in bone matrix, whereas the partial correlation analyses were conducted between the tissue‐level toughness of bone and GAGs or bound water when bound water or GAGs were used as covariate in the analysis, respectively. The statistical significance was considered only when *p* < 0.05.

## Results

### The age‐related decrease of the amount of GAGs in bone mineral matrix

**Figure 1:**
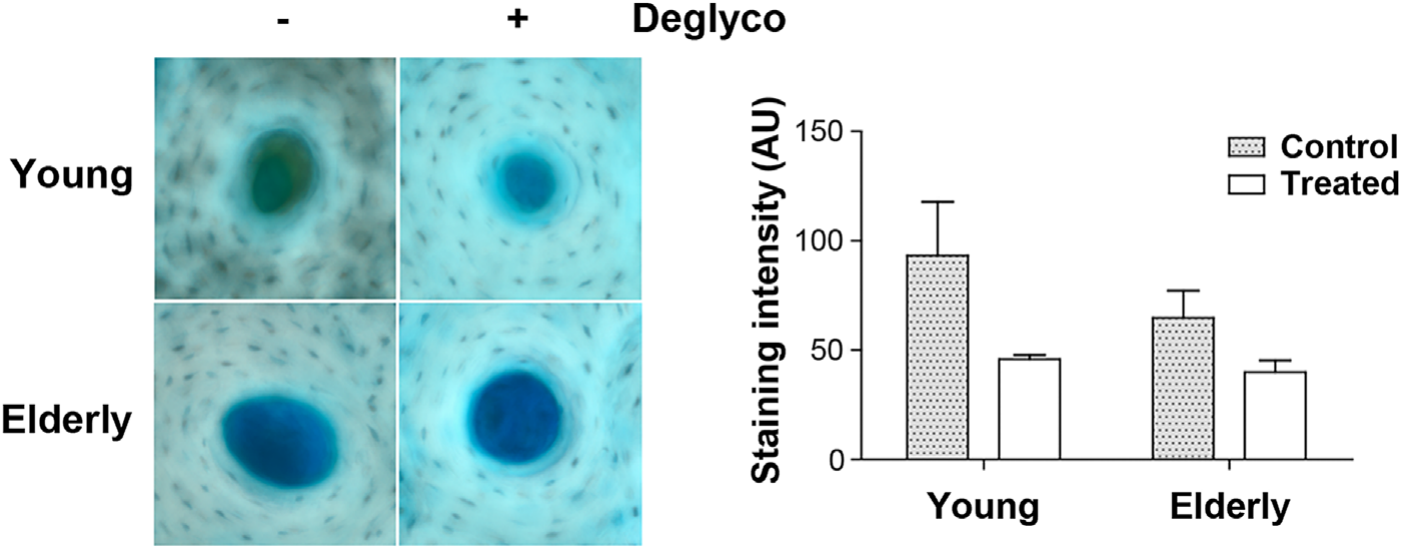
More GAGs in young than elderly human bone samples staining and deglycosylation assay. Cortical bone tissue sections from young and elderly donors were treated with or without Protein Deglycosylation Enzyme Mix and then histochemically stained with Alcian Blue (left panel). The intensity of the staining was quantified by NIH Image J software (right panel). The more dramatic reduction of GAGs by deglycosylation further confirmed the presence of more GAGs in young human bone samples. Data shown are mean ± SEM. *n* = 3.

The age‐dependent loss of GAGs was analyzed in young and elderly groups. Comparing the relative amount of GAGs between young and elderly human bone samples, a decrease of GAGs was observed in bone samples from the elderly group (Fig. 1). To further confirm the difference between the amount of GAGs in these two age groups, we removed the glycosylation modification using Protein Deglycosylation Enzyme Mix. The results showed that after the treatment, similar levels of GAGs were retained, suggesting that the level of GAGs was higher in young bone samples.

**Figure 2:**
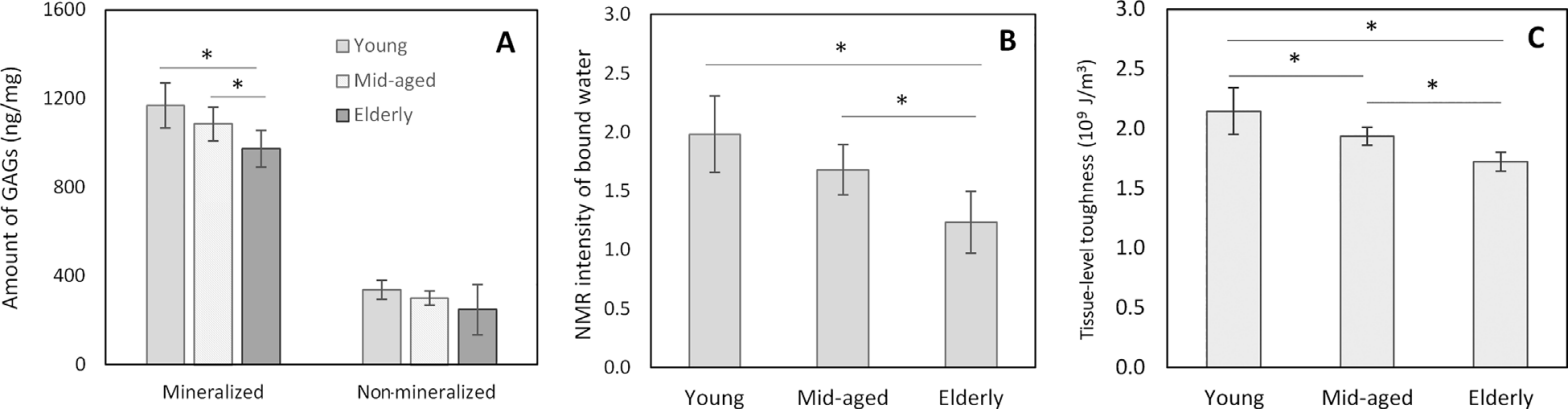
Age‐related effects on downregulation of the amount of GAGs (*A*), amount of bound water (*B*), and tissue‐level toughness of bone (*C*). There was a significant decrease of GAGs in the mineralized portion of bone, whereas no such decrease in GAGs was observed in the nonmineralized portion of bone matrix. Data shown are mean ± SD. *n* = 6. *p < 0.05.

The level of GAGs was determined both in mineralized and nonmineralized bones in this study. The experimental results showed that the GAGs were present mainly in the mineralized compartment, taking up almost 80% of the total GAGs in the bone matrix (Fig. 2 *A*). The amount of GAGs in the mineralized compartment of bone decreased with increasing age. The one‐factor ANOVA analysis of the results exhibited that the age‐related effect on the amount of GAGs was observed only in the mineralized compartment of bone (*p <* 0.05) but not in the nonmineralized compartment of bone (*p* > 0.05). The multiple comparisons indicated that both young and mid‐aged groups had significantly higher amounts of GAGs than the elderly group (*p* < 0.05).

### Age‐related effect on the amount of bound water and tissue‐level toughness of bone

One‐factor ANOVA analysis demonstrated that aging has a significant effect on the amount of bound water in bone measured using the low‐energy NMR approach (*p* < 0.05). The multiple comparisons revealed that such differences existed only between young versus elderly and mid‐aged versus elderly groups with no significant differences between the young and mid‐aged groups (Fig. 2 *B*).

One‐factor ANOVA analysis also exhibited that the tissue‐level toughness of bone, determined by nanoscratch test, was significantly affected by aging (*p* < 0.05). The multiple comparisons indicated that the significant differences (*p* < 0.05) existed between all aged groups (Fig. 2 *C*).

### Correlations among the tissue‐level toughness, GAGs, and bound water in bone

**Figure 3:**
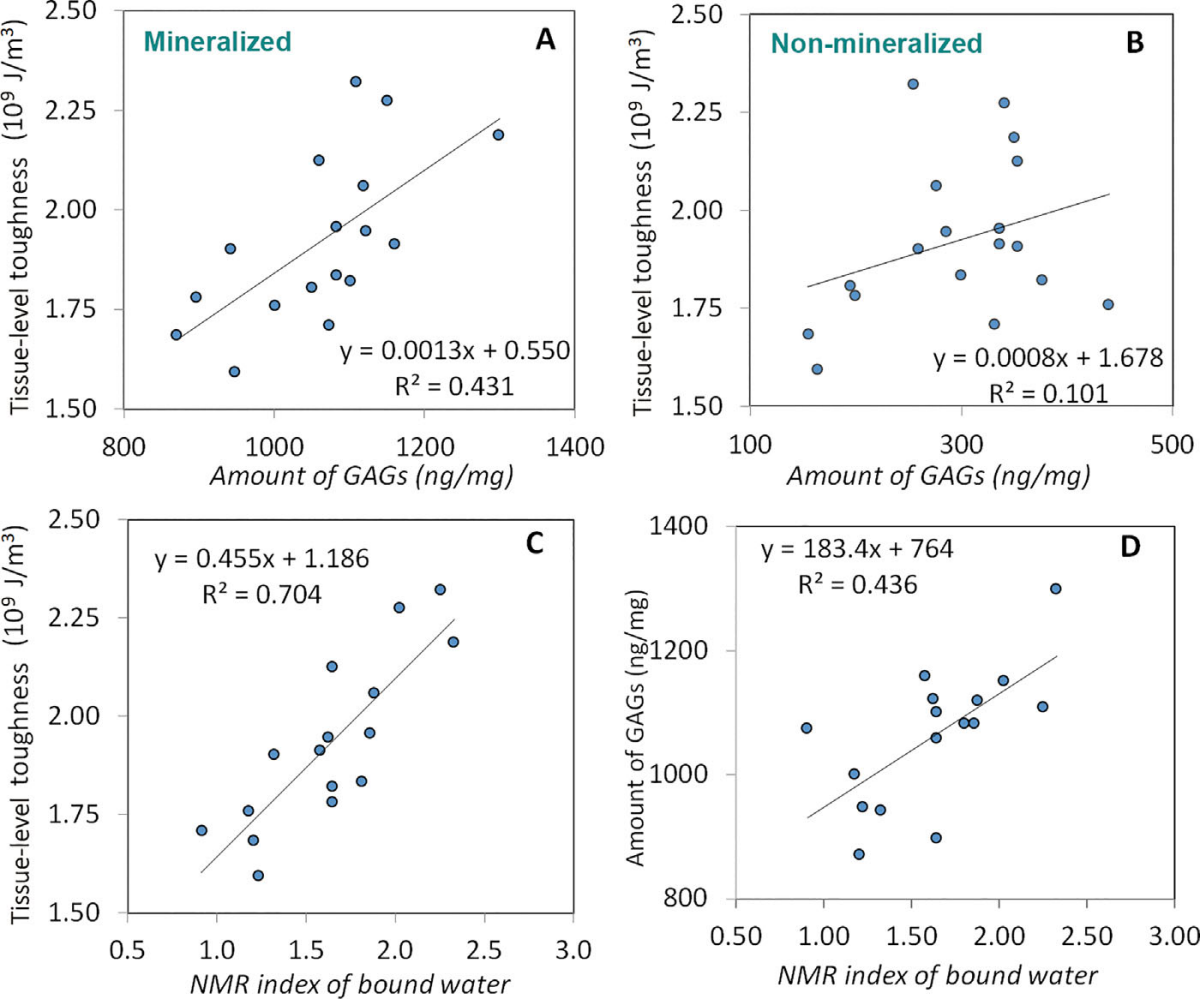
The correlations of tissue‐level toughness with GAGs and bound water in the mineralized portion of bone. Tissue‐level toughness of bone increased significantly (*p* < 0.05) with the amount of GAGs in the mineralized compartment (*A*) but had no statistically significant correlation (*p* > 0.05) with the nonmineralized portion of bone matrix (*B*). The correlations among the amount of bound water and the tissue‐level toughness (*C*) and the amount of GAGs (*D*) in bone. The analysis showed statistically significant and positive correlations between the parameters (*p* < 0.05).

The tissue‐level toughness of bone was significantly correlated (*p* < 0.05) with the amount of GAGs in the mineralized compartment but had no significant correlation (*p* > 0.05) with the GAGs in the nonmineralized compartment of bone matrix (Fig. 3 *A*, *B*). The tissue level toughness of bone increased as the amount of GAGs in the mineralized bone matrix increased.

A strong linear correlation (*p* < 0.05) was observed between the NMR index of bound water and the tissue‐level toughness of bone (Fig. 3 *C*). The tissue‐level toughness increased as the amount of bound water increased. There existed a significant correlation between the amount of bound water and GAGs (*p* < 0.05) in the mineralized bone matrix (Fig. 3 *D*). The amount of bound water increased as the amount of GAGs in bone matrix increased.

Pearson correlation analyses indicated that the amount of bound water, the amount of GAGs, and the tissue‐level toughness of bone were significantly correlated between each other (*p* < 0.05) (Table 1). The correlation between the tissue‐level toughness and amount of bound water was *r* ^2^ = 0.704 (*p* < 0.01). However, the partial correlation analysis revealed that the association of the tissue‐level toughness with bound water was reduced to *r* ^2^ = 0.515 when the effect of GAGs was removed from the analysis but still statistically significant (*p* < 0.05). On the other hand, the association of the tissue‐level toughness with GAGs was reduced from *r* ^2^ = 0.431 to *r* ^2^ = 0.05 when the effect of bound water was removed from the analysis, which was no longer statistically significant (*p* = 0.35). These results indicate that bound water is an independent factor, whereas GAGs impose effects on the tissue‐level toughness of bone via regulating the amount of bound water in bone matrix.

**Table 1:** Pearson Correlation Between GAGs, Bound Water, and the Tissue‐Level Toughness of Bone

	Amount of GAGs^a^	Amount of bound water	Tissue‐level toughness
Amount of GAGs	1.000	0.660^b^	0.659^b^
Amount of bound water	0.660^b^	1.000	0.841^b^
Bone tissue toughness	0.659^b^	0.841^b^	1.000

GAGs = glycosaminoglycans.

^a^ GAGs in the mineralized compartment only.

^b^ Correlation is significant at 0.01 level (two‐tail).

### Chondroitin sulfate (CS) is a major subtype of GAGs in the mineralized matrix of human bone

**Figure 4:**
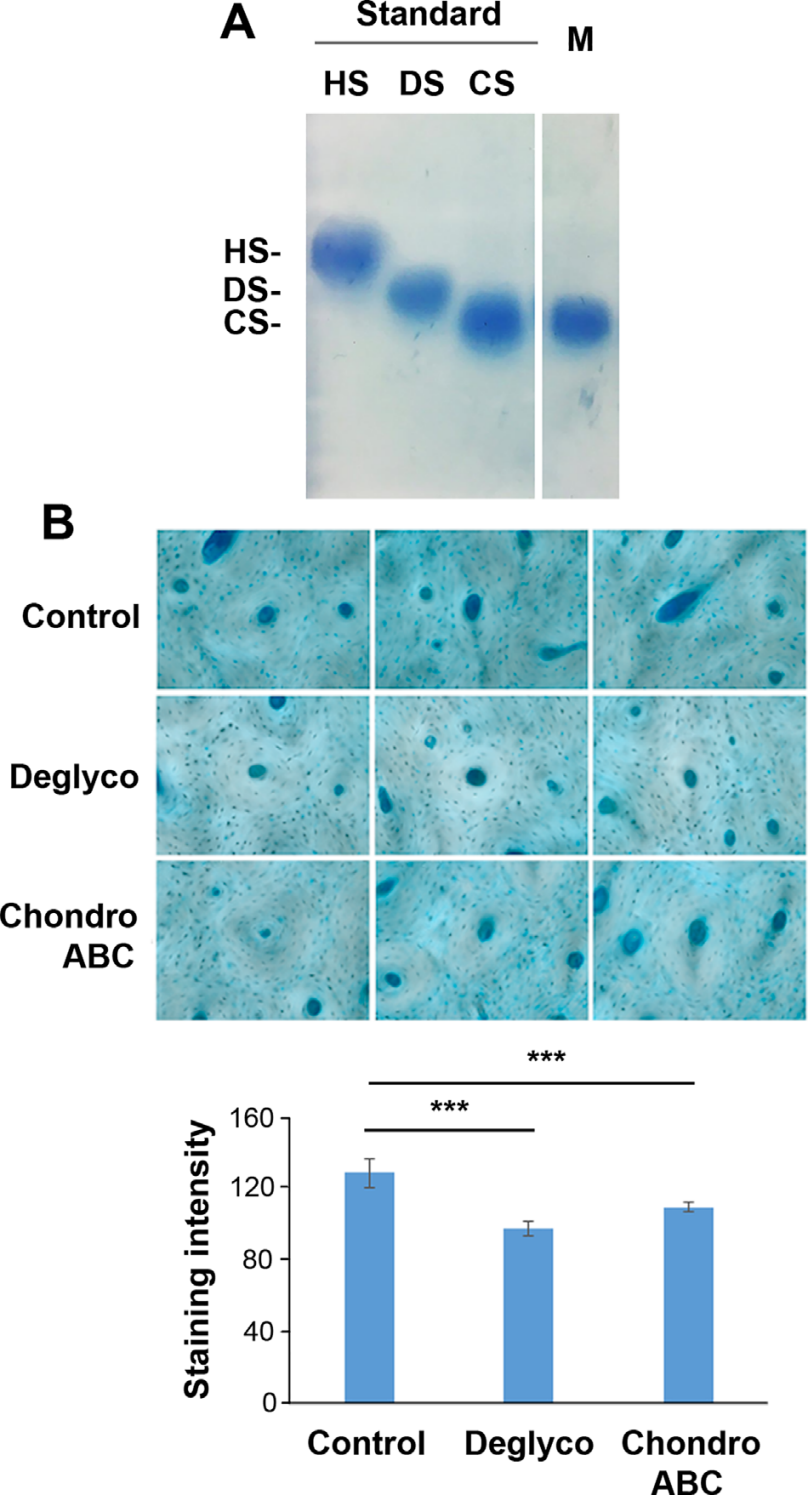
Chondroitin sulfate is a major GAG subtype in the mineral matrix of human bone. (*A*) Agarose gel electrophoresis showed various subtypes of GAGs, chondroitin sulfate (CS), dermatan sulfate (DS), and heparin sulfate (HS). CS was detected in mineralized (M) bone matrix. (*B*) Cortical bone tissue sections from young human subjects were treated with or without Protein Deglycosylation Enzyme Mix or chondroitinase ABC and then histochemically stained with Alcian Blue (top panel). The intensity of the staining was quantified by NIH Image J software (bottom panel). Data shown are mean ± SD. *n* = 6. ***p < 0.001.

Agarose electrophoresis assays demonstrated that only CS, but not heparin sulfate (HS) and dermatan sulfate (DS), was identified in the mineralized (M) compartment (Fig. 4 *A*). By treating bone samples with chondroitinase ABC, an enzyme that specifically removes CS and DS from PGs, Alcian blue staining and image‐based quantification showed that the treatment removed comparable levels of GAGs as that with Protein Deglycosylation Mix, which could remove any types of GAGs from core proteins (Fig. 4 *B*), suggesting that the majority of GAGs in mineralized matrix is in the form of CS.

### Age‐related decrease of CS in bone matrix

**Figure 5:**
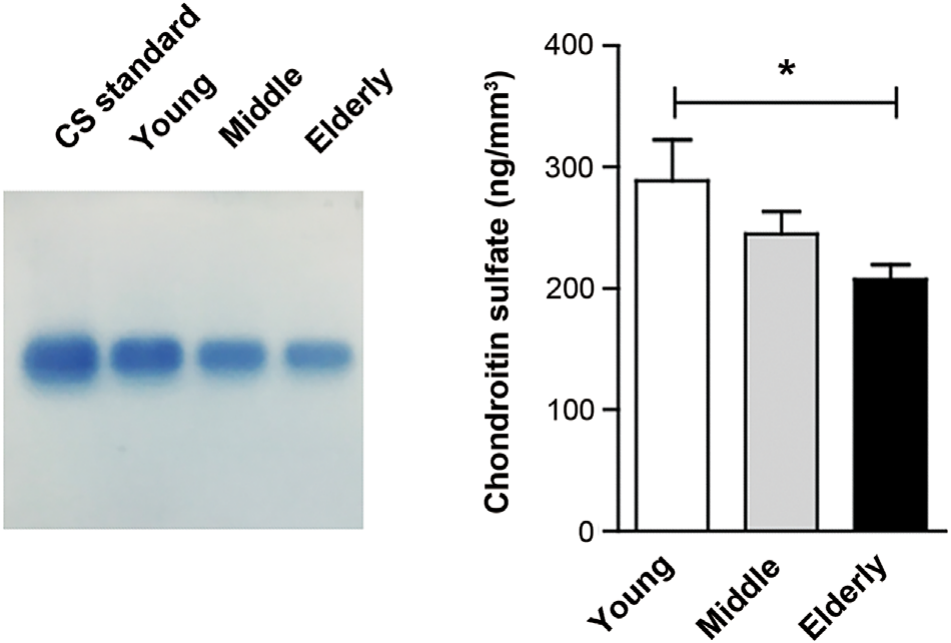
Chondroitin sulfate in human bone mineralized matrix is decreased during aging. PGs were isolated from human mineralized bone compartments and analyzed on agarose gel electrophoresis. The data showed age‐related reduction in the amount of CS in bone matrix. Data shown are mean ± SEM. *n* = 5–6. *p < 0.05.

Agarose electrophoresis assays and image‐based quantification further revealed that the amount of CS per bone volume decreased with age in the mineralized compartment of bone (Fig. 5). A statistically significant difference was observed between young and elderly groups (*p* = 0.046), whereas the difference between young and mid‐aged groups was not statistically significant. Nonetheless, the trend of age‐related decrease of CS in bone matrix was distinctively discernible.

### Age‐related decrease of biglycan in bone matrix

**Figure 6:**
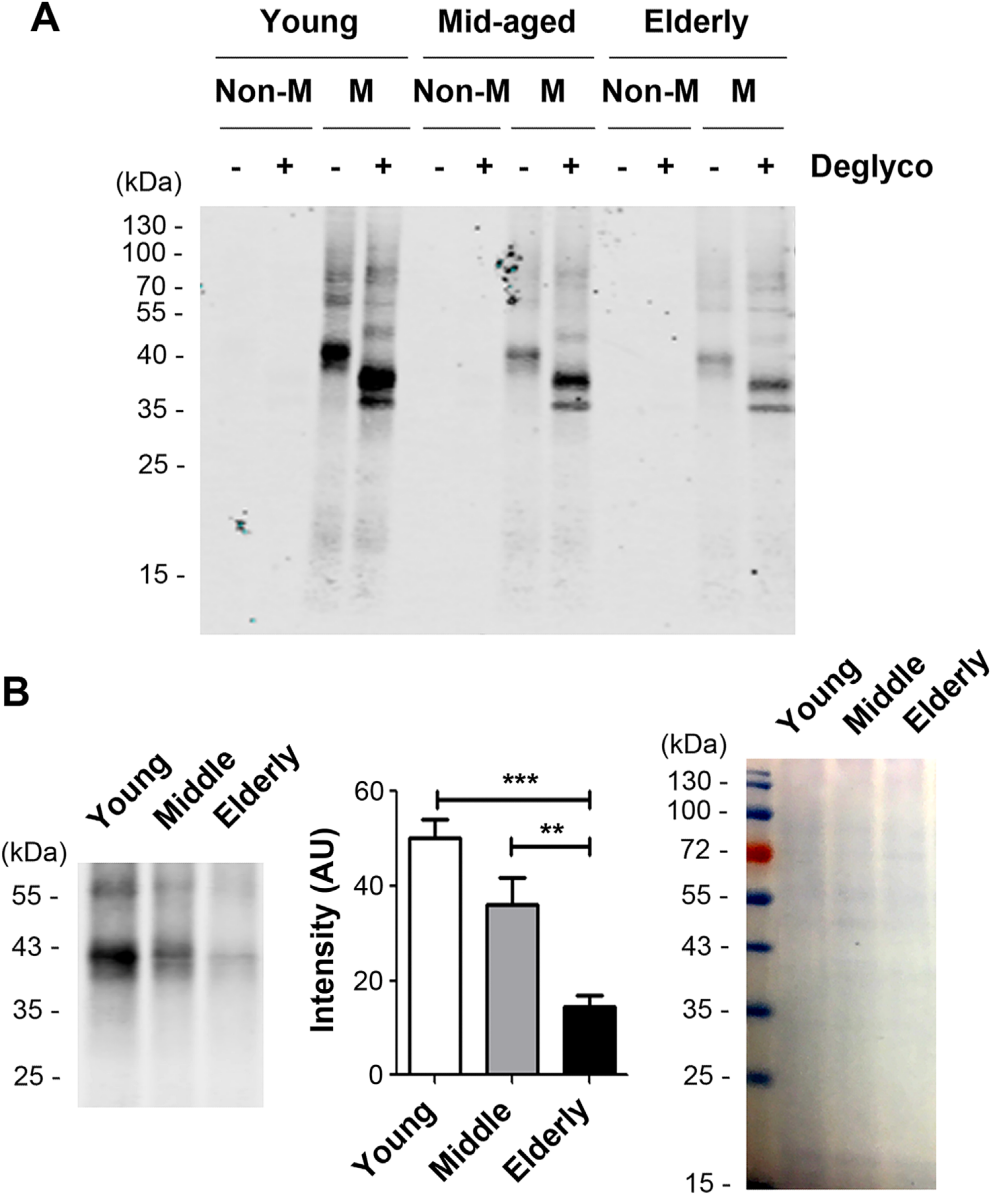
Biglycan is enriched in mineralized matrix of the bone and more abundant in young bones. Bone lysates were isolated from mineralized (M) or nonmineralized (Non‐M) compartments of young, mid‐aged, and elderly human bones. (*A*) The same amount of proteins in each sample was treated with or without Protein Deglycosylation Enzyme Mix to remove polysaccharide (Deglyco). The samples were loaded on SDS‐PAGE and immunoblotted with anti‐biglycan antibody. (*B*) The intensity of biglycan bands on immunoblots were quantified (middle). Total proteins loaded were stained with Coomassie Brilliant Blue (right panel). Data shown are mean ± SEM. *n* = 6. **p < 0.01; ***p < 0.001.

Biglycan is a major CS‐containing proteoglycan subtype in bone tissues.^52^, ^53^, ^54^, ^55^ Western blotting assay confirmed that the level of biglycan in mineralized matrix was age‐dependent, showing more abundant biglycan in young bones than in elderly ones (Fig. 6 *A*). Deglycosylation further confirmed the decrease of core biglycan protein levels associated with aging. Interestingly, biglycan was barely detectable in the nonmineralized compartment of bone. The relative levels of biglycan protein were further quantified and compared between aged groups (Fig. 6 *B*). A significant decrease of biglycan was observed in the elderly group compared with young and middle‐aged groups (left two panels) with comparable amounts of proteins loaded on the gel (right panel).

## Discussion

The results strongly support the hypothesis of this study that the amount of GAGs in bone matrix decreases with age and such changes may lead to significant reduction in the tissue‐level toughness of bone. This study reveals that the loss of bound water with aging is in great part attributable to the loss of GAGs in bone matrix with increasing age. In addition, this study demonstrates that CS, as a major subtype of GAGs, decreases with age. Correspondingly, biglycan, a major subtype of CS‐containing PGs, also declines in bone matrix.

Moreover, these results also suggest that age‐related reduction of biglycan is most likely a major reason for the diminishing amount of GAGs in bone with aging. There are two possible reasons: 1) biglycan level is much lower in aged bone compared with younger groups; 2) we detected CS as a major GAG subtype in mineralized matrix, which also decreased with aging. Because biglycan is a major CS‐containing PG in bone matrix, the age‐related reduction in GAGs is most likely attributable to the reduced amount of biglycan.

It has been well known that the bulk toughness of bone deteriorates with increasing age. Among the potential influencing factors, loss of bound water in bone matrix has been reported in previous studies to correlate strongly with the age‐related deterioration of bone toughness.^42^, ^43^ Based on the observation, magnetic resonance imaging (MRI) techniques are employed to determine the bound water content in bone matrix as a biomarker of bone fragility.^56^, ^57^, ^58^, ^59^ Nonetheless, the underlying mechanism is still poorly understood. This study, for the first time, indicates that loss of GAGs may be potentially one of the molecular origins of age‐related deterioration of bone quality via reducing the amount of bound water in the tissue.

GAGs, as part of PGs, are highly electrically charged and have great potential to attract and retain water in connective tissues (eg, articular cartilage).^60^, ^61^, ^62^ GAGs are also found in the extrafibrillar matrix of bone^21^, ^22^ with strong affinity to extrafibrillar minerals.^41^, ^63^, ^64^, ^65^ Collagen‐bound water is usually considered as a major source of organic‐bound water in bone matrix because type I collagen comprises 90% of the organic matrix of the tissue and is highly hydrophilic.^20^ However, bound water retained in the extrafibrillar matrix by NCPs (10% of the organic phase), which are highly hydrophilic too, may also significantly affect the mechanical behavior of bone. In fact, recent evidence shows that the effect of hydration on bulk mechanical properties of bone is also manifested in the mechanical response of the mineral phase,^32^, ^66^ suggesting that the hydration condition of extrafibrillar matrix is strongly associated with the bulk behavior of bone.^35^ It is not surprising because the extrafibrillar matrix, which is primarily composed of mineral crystals, takes up at least 23% of bone volume (assuming mineralized collagen fibrils are closely packed). Any changes in the extrafibrillar matrix would be reflected in the overall mechanical behavior of bone. By combining the results of previous studies and observed in this study, it could be reasonably conjectured that the hydration status in the extrafibrillar matrix, which is most likely regulated by the amount of GAGs in the matrix, has a significant effect on both tissue level and bulk properties of bone.

Because of the brittleness of mineral crystals and compliant nature of the collagen phase, the possible pathways for plastic deformation in bone would be limited to the sliding between mineral crystals,^67^ between mineral and collagen,^67^ and/or between the mineralized collagen fibrils.^68^ Using Raman spectroscopy and scanning electron microscopy, a previous study reveals that the ultrastructure of bone is adapted in both the organic and inorganic phases in response to mechanical loading and deformation, showing that a pressure‐induced shear band may form in the mineral phase of bone.^69^ More intriguingly, a recent study indicates that nanogranular friction between mineral particles in the extrafibrillar matrix rather than organic‐mineral bonding may be responsible for yielding of bone in compression, suggesting that the cohesion between the mineral crystallite (granules) most likely originates from the surrounding organic matrix.^70^ Indeed, it has been speculated that the plastic deformation in bone is originated through the relative sliding between mineral crystals.^67^, ^71^ Thus, it is presumable that the extrafibrillar matrix is directly involved in the plastic behavior of bone and GAGs may be a major player for the intergranular sliding between the mineral crystals.

Another interesting finding of this study is that the age‐related loss of GAGs occurs mainly in the mineralized bone matrix rather than in the nonmineralized compartments. Because osteoids (bone matrix in premineralization stage) are newly formed tissue in the nonmineralized compartment of bone, this result suggests that it is unlikely that age‐related changes in GAGs are induced during the initial process of bone formation but happen in the mineralized bone matrix as people age. Two possible scenarios could be considered: 1) GAGs may not be as abundant in newly formed tissue in elderly bone compared with the newly formed tissue in young bone; or 2) GAGs are lost in existing bone matrix through some unknown pathways in aged bone. To elucidate the mechanism, further investigations are needed.

Several previous reports have shown that biglycan is a major form of small PGs present in bone mineral matrix^53^, ^54^, ^55^ and is richly expressed in human bone tissue.^52^ It was found in this study that CS was likely a predominant subtype of GAGs in bone matrix. Moreover, both CS and CS‐associated biglycan showed significant decreases with age. These results are in good agreement with those reported in the literature.^72^, ^73^ Nonetheless, we cannot exclude the possible involvement of other subtypes of PGs and GAGs in bone matrix. Previous studies show that decorin (dcn) is another major subtype of PGs in bone matrix. Although Dcn‐deficient mice have no obvious phenotype in bone tissue properties possibly because of its redundant role with biglycan,^55^ double knockout mice have more severe phenotype than the single biglycan knockout.^74^ Thus, it is likely that biglycan along with other PGs may jointly possess the capability of retaining water in bone matrix. We will explore the specific function of the potential PGs using transgenic animal models in the future.

There are several limitations of this study. First, this study examined the effect of age‐related loss of GAGs only on the tissue‐level toughness of bone. This measure may directly reflect the change of bone mechanical properties at ultrastructural levels, and thus ought to be sufficient for testing the hypothesis of this study. Although previous study has shown that the tissue level toughness of bone measured using nanoscratch tests is strongly correlated with the bulk toughness of bone,^33^ further studies may still be necessary to determine the relative contribution of GAGs loss to the bulk mechanical properties compared with all other factors at different hierarchies of bone. Second, we used a semiquantitative Alcian blue staining method to measure relative contents of GAGs on bone tissue sections. This method has been used in other studies for quantifying GAG levels in bone sections^75^, ^76^ and in cultured cells.^77^, ^78^ This histochemical method was utilized to verify the results obtained from bone powder samples using enzyme digestion approaches. Because the powder samples were prepared manually, it was hard to keep the consistent size and shape distributions of the powders for each individual donor. Thus, the amount of GAGs in bone matrix was measured using both methods to avoid potential experimental errors. Third, only CS and biglycan were investigated in this study. Several previous reports have shown that biglycan is a major form of small PGs present in bone mineral matrix^53^, ^54^, ^55^ and is richly expressed in human bone tissue.^52^ In this study, we did not observe predominant presence of other subtypes of GAGs. However, we could not exclude the involvement of other possible subtypes of PGs at minor presence in current study. Aging may have an effect on the other subtypes of GAGs and PGs, such as keratin sulfate and dcn, which are also reported to reside in bone matrix. As discussed above, this will be the next research topic of our group.

In conclusion, this study indicates that loss of GAGs, which subsequently results in loss of bound water in the matrix, is potentially one of the molecular origins of age‐related deterioration of bone quality. In addition, this study shows that CS is a major subtype of GAGs and CS‐containing biglycan in bone matrix decreases with aging, thus resulting in loss of GAGs in bone matrix.

## Disclosures

All authors state that they have no conflicts of interest.
